# Mechanisms for cardiac calcium pump activation by its substrate and a synthetic allosteric modulator using fluorescence lifetime imaging

**DOI:** 10.1093/pnasnexus/pgad453

**Published:** 2023-12-22

**Authors:** Jaroslava Šeflová, Carlos Cruz-Cortés, Guadalupe Guerrero-Serna, Seth L Robia, L Michel Espinoza-Fonseca

**Affiliations:** Department of Cell and Molecular Physiology, Loyola University Chicago, Maywood, IL 60153, USA; Center for Arrhythmia Research, Department of Internal Medicine, Division of Cardiovascular Medicine, University of Michigan, Ann Arbor, MI 48109, USA; Center for Arrhythmia Research, Department of Internal Medicine, Division of Cardiovascular Medicine, University of Michigan, Ann Arbor, MI 48109, USA; Department of Cell and Molecular Physiology, Loyola University Chicago, Maywood, IL 60153, USA; Center for Arrhythmia Research, Department of Internal Medicine, Division of Cardiovascular Medicine, University of Michigan, Ann Arbor, MI 48109, USA

## Abstract

The discovery of allosteric modulators is an emerging paradigm in drug discovery, and signal transduction is a subtle and dynamic process that is challenging to characterize. We developed a time-correlated single photon-counting imaging approach to investigate the structural mechanisms for small-molecule activation of the cardiac sarcoplasmic reticulum Ca^2+^-ATPase, a pharmacologically important pump that transports Ca^2+^ at the expense of adenosine triphosphate (ATP) hydrolysis. We first tested whether the dissociation of sarcoplasmic reticulum Ca^2+^-ATPase from its regulatory protein phospholamban is required for small-molecule activation. We found that CDN1163, a validated sarcoplasmic reticulum Ca^2+^-ATPase activator, does not have significant effects on the stability of the sarcoplasmic reticulum Ca^2+^-ATPase–phospholamban complex. Time-correlated single photon-counting imaging experiments using the nonhydrolyzable ATP analog β,γ-Methyleneadenosine 5′-triphosphate (AMP-PCP) showed ATP is an allosteric modulator of sarcoplasmic reticulum Ca^2+^-ATPase, increasing the fraction of catalytically competent structures at physiologically relevant Ca^2+^ concentrations. Unlike ATP, CDN1163 alone has no significant effects on the Ca^2+^-dependent shifts in the structural populations of sarcoplasmic reticulum Ca^2+^-ATPase, and it does not increase the pump's affinity for Ca^2+^ ions. However, we found that CDN1163 enhances the ATP-mediated modulatory effects to increase the population of catalytically competent sarcoplasmic reticulum Ca^2+^-ATPase structures. Importantly, this structural shift occurs within the physiological window of Ca^2+^ concentrations at which sarcoplasmic reticulum Ca^2+^-ATPase operates. We demonstrated that ATP is both a substrate and modulator of sarcoplasmic reticulum Ca^2+^-ATPase and showed that CDN1163 and ATP act synergistically to populate sarcoplasmic reticulum Ca^2+^-ATPase structures that are primed for phosphorylation. This study provides novel insights into the structural mechanisms for sarcoplasmic reticulum Ca^2+^-ATPase activation by its substrate and a synthetic allosteric modulator.

**Significance Statement**

Allosteric modulation is a growing concept in drug discovery, yet unraveling the structural mechanisms that underlie this phenomenon continues to pose a challenge. We developed a time-correlated single photon-counting (TCSPC) imaging approach to investigate the structural mechanisms for allosteric activation of the cardiac calcium pump sarcoplasmic reticulum Ca^2+^-ATPase (SERCA2a), a pharmacological target for the treatment of heart failure. We established that ATP, the molecule that fuels SERCA2a, is both a substrate and modulator of the pump and showed that a synthetic allosteric activator enhances the ATP-mediated modulatory effects to populate SERCA2a structures that are ready for SERCA2a phosphorylation. This study shows the synergy between SERCA2a's substrate and a synthetic allosteric modulator to activate a clinically important target in the heart.

## Introduction

The concept of allostery for drug discovery is a growing paradigm in medicinal chemistry (1, 2). In this paradigm, allosteric drugs modulate the activity through the propagation of allosteric signaling, producing either inhibition or activation of the target. Understanding the structural mechanisms for small-molecule modulation is an essential prerequisite for allosteric drug discovery (3), but signal transduction is a dynamic process involving subtle structural changes (4, 5) that are challenging to characterize using traditional experimental techniques (6). This motivates the development of novel approaches to determine the structural mechanisms underlying small-molecule allosteric modulation of druggable proteins.

The cardiac calcium pump (sarcoplasmic reticulum Ca^2+^-ATPase, SERCA2a) plays an essential role in normal cardiac function, clearing cytosolic Ca^2+^ needed to relax muscle cells in each heartbeat (diastole) (7). SERCA pumps two Ca^2+^ ions into the sarcoplasmic reticulum (SR) lumen using energy derived from the hydrolysis of ATP (8, 9) and is regulated by the 52-residue membrane protein phospholamban (PLN). PLN regulates SERCA2a by inhibiting its ability to transport Ca^2+^ (10, 11), and inhibition is relieved by phosphorylation of PLN (12–16). A key molecular dysfunction in patients with heart failure usually involves insufficient SERCA expression and impaired PLN phosphorylation, leading to SERCA2a inactivation and decreased Ca^2+^ transport in the cardiomyocyte. Reactivation of Ca^2+^ transport results in improved cardiac function in experimental models of heart failure (17–21), and SERCA2a is a well-validated target and its small-molecule activation is a promising approach for heart failure therapy (22, 23).

SERCAs structure, function, and regulation are the subjects of hundreds of papers (see recent reviews) (24–28) yet there is not a single study addressing the mechanisms for the small-molecule activation of this pump. In addition, there are no crystal structures of SERCA bound to small-molecule activators, so the structural basis for small-molecule SERCA activation remains enigmatic. This has limited systematic, structure-based drug discovery and hit-to-lead optimization campaigns aimed at the discovery of therapeutic products targeting this pharmacologically important pump. In this study, we introduce a time-correlated single photon counting (TCSPC) imaging method to investigate in unprecedented detail the mechanisms for small-molecule SERCA2a activation. This approach revealed novel features of small-molecule activation of SERCA2a, including the concept that SERCA2a activation by a small-molecule effector does not require dissociation of the SERCA2a–PLN complex, the notion that ATP is both a substrate and an effector of the pump and that stimulation of SERCA2a arises from the synergy between the activator and ATP to populate structures primed for SERCA2a phosphorylation. The result is a vivid visualization of the molecular mechanism for the small-molecule activation of a druggable target in the heart.

## Materials and methods

### Chemicals

All chemicals used in this study were purchased at reagent quality (purity > 95% by high-performance liquid chromatography): CDN1163, *N*-(2-methylquinolin-8-yl)-4-propan-2-yloxybenzamide (Sigma, St. Louis, MO, USA); istaroxime, (3*E*,5*S*,8*R*,9*S*,10*R*,13*S*,14*S*)-3-(2-aminoethoxyimino)-10,13-dimethyl-1,2,4,5,7,8,9,11,12,14,15,16-dodecahydrocyclopenta[a]phenanthrene-6,17-dione (MedChemExpress LLC, Monmouth Junction, NJ, USA); CP-154526, *N*-butyl-*N*-ethyl-2,5-dimethyl-7-(2,4,6-trimethylphenyl)pyrrolo[2,3-d]pyrimidin-4-amine (Sigma), Ro 41-0960, (3,4-dihydroxy-5-nitrophenyl)-(2-fluorophenyl) methanone (Sigma); AMP-PCP, β, γ-methyleneadenosine-5′-triphosphate (Sigma).

### Isolation of enriched SERCA2a microsomes

Pig hearts were obtained after euthanasia and placed in a cardioplegic solution (280 mM glucose, 13.44 mM KCl, 12.6 mM NaHCO_3_, and 34 mM mannitol). Left ventricles free walls were obtained, minced, and homogenized with a cold buffer that contained 9.1 mM NaHCO_3_, 0.9 mM Na_2_CO_3,_ and a cocktail of proteases inhibitors (Sigma); the mixture was centrifuged at 6,500 × *g* for 30 min at 4 °C to remove debris. The supernatant was filtered, collected, and centrifuged at 14,000 × *g* for 30 min at 4 °C. The collected filtrate was centrifuged at 47,000 × *g* for 60 min at 4 °C. The pellet was resuspended in a solution containing 0.6 M KCl and 20 mM Tris (pH = 6.8). The suspension was centrifuged at 120,000 × *g* for 60 min at 4 °C, and the pellet was resuspended in a solution containing 0.3 M sucrose, 5 mM 3-(N-Morpholino)propanesulfonic acid (MOPS), and protease inhibitors (pH = 7.4). The protein concentration of the SR microsomal fraction was determined using the Pierce Coomassie plus assay kit (Thermo-Fisher Scientific, Waltham, MA, USA). The microsomal membranes were aliquoted, frozen in liquid nitrogen, and stored at −80 °C. SERCA2a purification was performed as described by Sitsel et al. (29).

### Western blot analysis

Samples were loaded into 4–20% Tris–Glycine polyacrylamide precast gels (ThermoFisher Scientific, Waltham, MA, USA) and electrophoresis was carried out. The electrophoresis under denaturing conditions (sodium dodecylsulphate polyacrylamide gel electrophoresis, SDS-PAGE) resolved proteins were transferred to iBlot stacks with regular polyvinylidene fluoride (PVDF) membranes using the iBlot 2 dry blotting system (ThermoFisher Scientific, Waltham, MA USA). Nonspecific binding sites were blocked with 5% nonfat dry milk in phosphate-buffered saline with tween (PBS-T) (in mmol/L, 3 KH_2_PO_4_, 10 Na_2_HPO_4_, 150 NaCl, and 0.15% Tween 20, pH 7.2–7.4) for 30 min at room temperature. Membranes were then incubated with specific primary antibodies for SERCA2a (1:500; ThermoFisher) and PLN (1:5000; Badrilla, UK), diluted in 5% bovine serum albumin in PBS-T overnight at 4 °C. After washing three times for 10 min, membranes were incubated with horseradish peroxidase-conjugated secondary antibodies, diluted in 5% bovine serum albumin in PBS-T. After washing three times for 10 min, protein-antibody reactions were detected using Pierce SuperSignal Chemiluminescent Substrates (ThermoFisher Scientific, Waltham, MA USA). We performed detection and quantification of protein bands with a Bio-Rad ChemiDoc system and Image Lab software 5 (Bio-Rad, Hercules, CA USA).

### SERCA ATPase activity assays

We performed SERCA2a activity assays using an enzyme-coupled β-Nicotinamide adenine dinucleotide (NADH)-linked ATPase assay described previously (30). Briefly, we measured the activity of Ca^2+^ ATPase in µmol min^−1^ mg^−1^ from the decrease in absorbance of NADH at 340 nm at 25 °C in a 96-well format using a Synergy H1 (BioTek, Winooski, VT) microplate reader. Each well contained a 200 µL final volume of assay buffer containing SERCA buffer (50 mM MOPS, 100 mM KCl, 5 mM MgCl_2_, and 1 mM ethylene glycol-bis(2-aminoethylether)-N,N,N′,N′-tetraacetic acid (EGTA), pH = 7), 5U lactate dehydrogenase, 5U pyruvate dehydrogenase, 1 mM phosphoenolpyruvate, 5 mM ATP, 0.2 mM NADH, 2 µg of microsomal suspension, 2 µM of Ca^2+^ ionophore A23187, and eight free Ca^2+^ concentrations. Each concentration of the compounds tested here was calculated to a final volume of 200 µL. We incubated the small molecules for 30 min at 25 °C with the reaction mixture. Concentration-response curves for each compound were constructed with the data from [Ca^2+^]-dependent SERCA activity curves performed at compound concentrations of 0.1–100 µM. The final free Ca^2+^ concentrations were calculated using MaxChelator (31), and were achieved by using twelve individual stock CaCl_2_ solutions. Each plate included untreated and thapsigargin (TG)-treated microsomes as controls. To account for biological variability, we use microsomal fractions obtained from three pig hearts. In all cases, the maximal activity values (*V*_max_) were normalized relative to the compound-free samples.

### HEK-293T cell culture

**Fig. 1.**
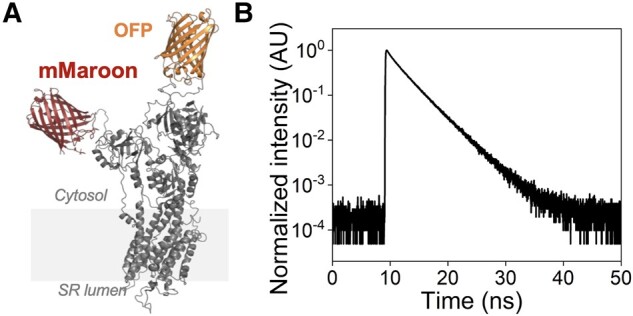
Overview of the TCSPC imaging approach to probe the mechanisms for SERCA2a activation. A) Snapshot of the modeled 3D structure of the two-color cardiac SERCA2a construct labeled with a maroon fluorescent protein (mMaroon) at position Met1 and with an orange fluorescent protein (OFP) at position Gly509. The approximate location of the lipid bilayer is represented by the shaded box. We note that this structure is shown for representation only. B) Fluorescence decay measured by TCSPC for two-color SERCA2a in the DMSO vehicle. We found that the TCSPC imaging approach has over 4 orders of magnitude difference between signal (intensity at ∼10^0^, arbitrary units (AU)) and noise (intensity at ∼10^−4^ AU).

The human two-color SERCA2a construct was produced as described previously (32). The mCerulean at the N-terminus of human SERCA2a was replaced by red-shifted orange fluorescence protein (mCyRFP1) (33) and yellow flourescent protein (YFP) was replaced by mMaroon1 (34) at the position before residue number 509 within the N-domain of SERCA2a (Fig. 1A). For microsomes containing SERCA2a and PLN, the mCyRFP1 and mMaroon were fused to the N-termini of SERCA2a and PLN (35), respectively. The mCyRFP1-mMaroon1 construct has a Förster distance of 63.34 Å (33, 36). The final constructs were verified by full-length sequencing (ACGT, USA). HEK 293T cells (CRL-3216, ATCC) were seeded into a 150 mm cell culture dish 72 h before transient transfection and cultured in Dulbecco’s modified Eagle’s medium (DMEM) (Corning, USA) supplemented with 10% fetal bovine serum (FBS) in 5% CO_2_ incubator at 37 °C. When cells reached 70–80% confluence, the cell culture media was replaced by fresh media, and cells were transfected with two-color SERCA construct (32, 36) using a Lipofectamine 3000 kit (Invitrogen, Life Technologies). Cells were harvested 48 h post-transfection and microsomal membranes were prepared on the day of the harvest.

### Microsomal membrane preparation from transfected HEK-293T cells

The microsomal membranes from HEK-293T were prepared according to the protocol published previously (32, 37). Specifically, each 150 mm cell culture dish was scraped into 12 mL of homogenization buffer (0.5 mM MgCl_2_, 10 mM Tris–HCl; pH 7.5) supplemented with UltraCruz protease inhibitor cocktail tablet without ethylenediaminetetraacetic (EDTA) (Santa Cruz Biotechnology). Cells were pelleted using centrifugation of 1,000 × *g* for 10 min at 4 °C and subsequently dissolved in 5 mL of fresh homogenization buffer. The cell suspension was homogenized with 20 strokes of Potter-Elvehjem glass homogenizer, and 5 mL of sucrose buffer (100 mM MOPS, 500 mM sucrose; pH = 7.0 with protease inhibitors). The crude homogenate was passed 10 times through a 27-gauge needle. Subsequently, the homogenate was centrifuged at 1,000 × *g* for 10 min at 4 °C to remove unbroken cells, mitochondria, and cellular debris. The supernatant was subjected to ultracentrifugation at 126,000 × *g* for 30 min at 4 °C, and the pellet was dissolved in a 1:1 mixture of homogenization and sucrose buffers. The total protein concentration was determined using bicinchoninic acid (BCA) assay (Pierce BCA Protein assay, ThermoFisher Scientific, Rockford, IL). The microsomal fractions were separated into single-use aliquots containing 50 μL of microsomal membranes at a concentration of 3–4.5 mg/mL. We performed four independent transfections to account for biological variability as part of our experimental design, so each experiment represents a biological replicate.

### Microsomal membrane preparation from HEK-293T cells infected with adenovirus

The 90% confluent cells were infected with human type 5 (dE/E3) adenovirus encoding two-color SERCA (Vector Biolabs, USA). The multiplicity of infection was 35. This value was experimentally determined using a small-scale infection. Infected cells were harvested 48 h post-transfection and microsomal membranes were prepared using the same methodology as described for transiently transfected HEK-293T cells.

### PLN-CDN1163 competition experiments

The SERCA2a competition experiment monitored Förster resonance energy transfer (FRET) changes between mCyRFP1-SERCA2a and mMaroon1-PLN microsomes. We used a physiologically relevant SERCA-to-PLN DNA ratio of 1:5 (38), and tested the effects of CDN1163 at increasing concentrations of the compound (0.1–50 μM). The donor alone lifetime was shortened to 2.63 ns due to FRET. This corresponds to average FRET efficiency of 26% using the following equation $\mathrm{FRET}\;\mathrm{Efficiency}\;(\text{\%})=100*\left(1-\frac{\tau_{DA}}{\tau_{D}}\right)$, where τ*_D_* is the lifetime of donor alone and τ*_DA_* is the lifetime of the donor in the presence of acceptor.

### Time-correlated single-photon-counting imaging of SERCA2a

Aliquots of microsomes were thawed on ice, mixed with the dimethylsulfoxide (DMSO) vehicle (0.01%) or 10 µM CDN1163 (>98% purity by HPLC; Sigma-Aldrich), and incubated for 30 min at room temperature. Subsequently, the microsomes were mixed with a buffer composed of 120 mM potassium aspartate, 15 mM KCl, 5 mM KH_2_PO_4_, 0.75 mM MgCl_2_, 2% dextran, 20 mM 4-(2-hydroxyethyl)piperazine-1-ethanesulfonic acid (HEPES), 2 mM EGTA, and CaCl_2_ 1.7 mM; pH 7.2 with or without 500 μM AMP-PCP and imaged immediately after mixing. The free [Ca^2+^] concentrations were estimated to range from 0.001 to 100 µM. The final free Ca^2+^ concentrations were calculated using MaxChelator (31), and were achieved by using twelve individual stock CaCl_2_ solutions. Time-correlated single-photon-counting (TCSPC) experiments were performed as previously described (35, 39). Donor (mCyRFP1) fluorescence in the membrane microsomes was excited by a supercontinuum laser beam (Fianium, Southampton, UK). A 482/18 nm bandpass filter was used for selective excitation of the FRET donor. Fluorescence emission was detected using a 525/50 nm emission bandpass filter. The laser was placed inside a drop of suspended microsomes, yielding a photon counting rate of 100,000 photons/s. Under those conditions, we observed less than 10% photobleaching during the 60 s of acquisition time. Fluorescence was detected through a 1.2 numerical apperture (NA) water-immersion objective with a photomultiplier assembly (PMA) hybrid detector (PicoQuant, Germany) connected to a single photon-counting module (HydraHarp 300, PicoQuant, Germany) with a time channel width of 16 ps. The TCSPC imaging method has over four orders of magnitude difference between signal and noise (Fig. 1B), making it ideal for capturing the subtle structural changes associated with allosteric modulation.

### Global analysis of the fluorescence lifetimes

Fluorescence decay histograms from four different sets of microsomes were analyzed in SymPhoTime 64 software (Picoquant, Germany) with the TCSPC global fitting tool. The donor alone (mCyRFP1-SERCA2a) showed slightly two-exponential decay with relative amplitude of the second component accounting for <8%, therefore, the donor alone lifetime was estimated as a single exponential decay with a lifetime of 3.56 ns for all tested conditions. The donor in the presence of the acceptor (two-color SERCA2a or mMaroon1-PLN) was best fitted using a two-exponential decay, where the amplitude-weighted average lifetime (τ_avg_) was shorter than the lifetime of the donor alone that is consistent with FRET between donor and acceptor. We performed fitting with the increasing complexity of the used model (increasing number of exponential functions used in the fit) and we evaluated the residual distribution of the performed fit and χ^2^. Therefore, the two-exponential model was selected as the correct model describing the decay of two-color SERCA2a. Each of the fitted exponential models corresponds to one component that is present in the overall decay and % content of this component. In addition, we performed two-exponential fitting of all experimental conditions and determined changes in both individual lifetimes. The values of these two individual lifetimes did not vary significantly within all tested groups which is suggestive of two structural species that change their relative % content (populations of fluorescent species). These changes in the populations are reflected as changes in the amplitude-weighted average lifetimes. We further performed a global fitting that allows us to share selected lifetimes within all experimental groups which eliminates variability in the mathematical model. We assume the presence of two populations of SERCA2a, open and closed, that are resolved by TCSPC, which agrees with our recent work (37).

### Statistical analysis

All results are presented as mean ± standard error of the mean (SEM). Significance was evaluated using the Mann–Whitney *U* test for paired experiments or a two-way analysis of variance (ANOVA) followed by Dunnett's post hoc test to analyze differences between the control and multiple treatments. We used 95% confidence intervals around the differences between the groups for the post hoc test. Two-sided *P* values were used, and α-level <0.05 was considered significant.

## Results

### ATPase assays validate the synthetic molecule CDN1163 as a SERCA2a activator

**Fig. 2.**
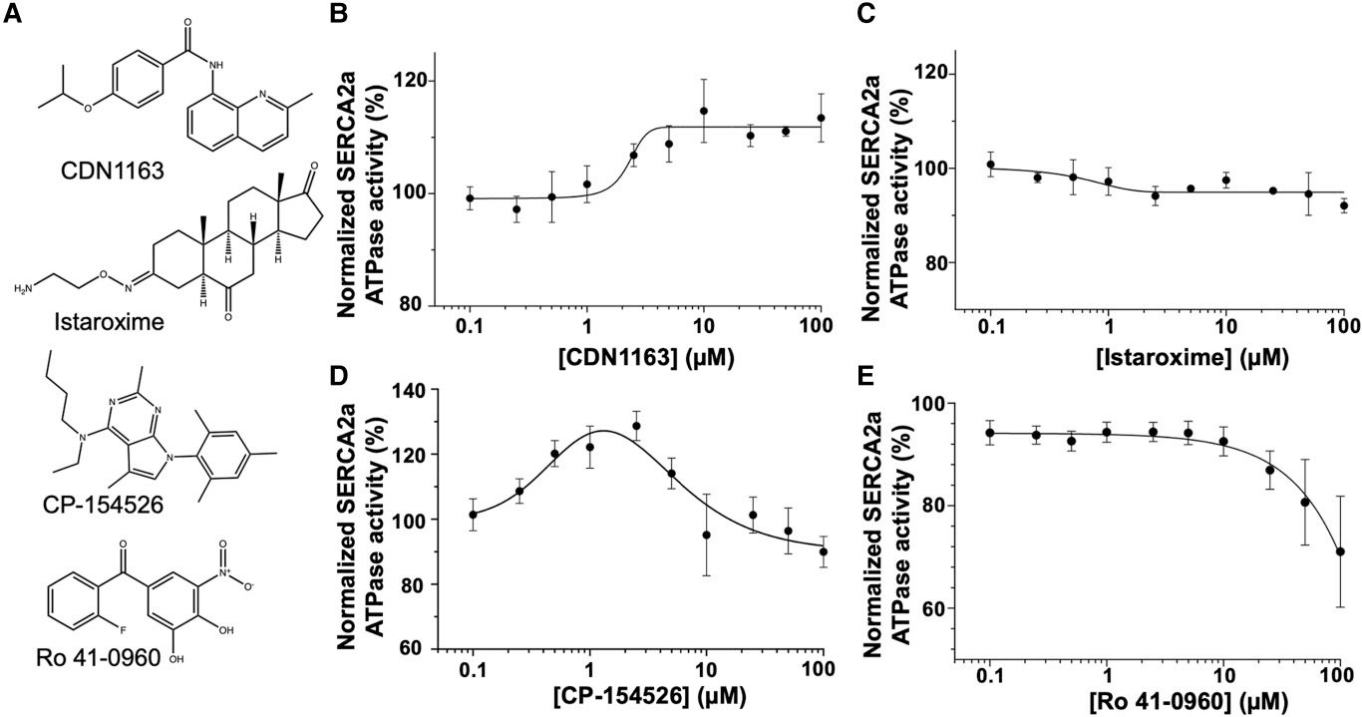
Effects of small-molecule effectors on SERCA2a ATPase activity. A) Structures of chemically diverse small-molecule effectors reported in the literature as SERCA activators. Ten-point concentration-response curves were obtained for B) CDN1163, C) istaroxime, D) CP-154526, and E) Ro 41-0960 on the ATPase activity of the cardiac SERCA2a. In all cases, the activity of the compounds at each concentration was obtained from an eight-point free Ca^2+^ concentration-dependent SERCA2a activity assay and normalized relative to the untreated control, as described in the experimental procedures. Data are reported as average ± SEM of three biological replicates (*N* = 3).

There are only a handful of small molecules that have been reported to stimulate SERCA activity: CDN1163, an allosteric activator that stimulates the nonmuscle SERCA isoform SERCA2b (40, 41); istaroxime, a molecule that was proposed to stimulate SERCA2a and inhibits the Na^+^/K^+^-ATPase (NKA) (42); CP-154526, which was reported to increase the maximal activity of muscle SERCA isoforms (43); and Ro 41-0960, a small molecule that increases both the activity and Ca^2+^ affinity of the SERCA2a pump (43) (Fig. 2A). The full activation profiles of these molecules have not yet been reported in the literature, therefore, we first used ATPase activity assays to establish the concentration-response profiles of these compounds on SERCA2a. In all cases, the SERCA2a activity is reported as the change of *V*_max_ relative to the activity of SERCA2a alone. When describing the data, we distinguish between “observed” data (i.e. data points) and “estimated” data (i.e. values derived from the fitted model data).

CDN1163 activates SERCA2a in a concentration-dependent manner with an EC_50_ value of 2.3 µM, and a maximal increase in the relative *V*_max_ activity of 11.8% estimated from the fitted curve (Fig. 2B). Notably, we found that the stimulatory effect of CDN1163 on SERCA2a remains constant at compound concentrations >10 µM (Fig. 2B). In our hands, istaroxime does not activate SERCA2a at any compound concentration (Fig. 2C). At high concentrations, istaroxime has a slight inhibitory effect on SERCA2a, however, this effect is not significant. We speculate that istaroxime does not activate SERCA2a in our assays, probably because of species-specific differences (44). However, we note additional ATPase assays performed by us showed that istaroxime inhibits the cardiac isoform of the a_1_ isoform of pig NKA-α_1_ with an IC_50_ = 0.47 µM; this IC_50_ value is virtually identical to that previously reported using the dog NKA-α_1_ (IC_50_ = 0.43 µM) (45). These findings suggest that, unlike CDN1163, istaroxime may be a species-specific activator of the SERCA2a pump. CP-154526 also activates SERCA2a in the high nM to low µM range, with an observed maximal increase in *V*_max_ of 28% at a compound concentration of 2.5 µM (Fig. 2D). However, this compound follows a bell-shaped dose-response curve, where increasing compound concentration leads to increased activity up to a point and further increases in compound concentration lead to decreasing or abolished activity (46). This effect is likely the result of CP-154526 aggregation, as suggested before by atomistic molecular dynamics simulations (47). The ATPase activation assays showed that Ro 41-0960 does not stimulate the activity of SERCA2a in the concentration range tested here (0.1–100 µM, Fig. 2E). Instead, Ro 41-0960 has an inhibitory activity at compound concentrations ≥25 µM, with an observed decrease in *V*_max_ by ∼30% at a compound concentration of 100 µM (Fig. 2E). In summary, only CDN1163 activates SERCA2a in a concentration-response manner, hence we used this effector as a molecular probe to study the mechanisms for SERCA2a activation.

### CDN1163 does not dissociate the SERCA–PLN complex

**Fig. 3.**
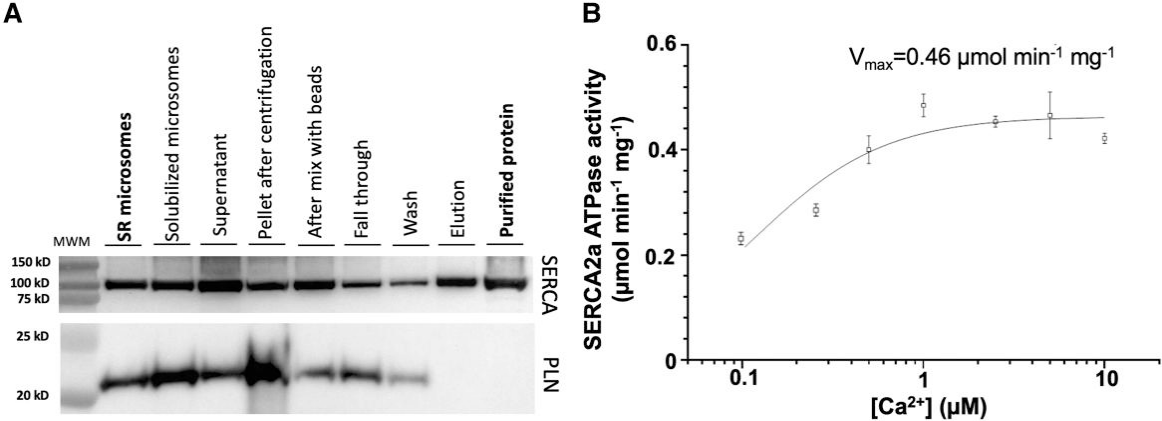
PLN removal does not affect the catalytic turnover of SERCA2a. A) Western blot analysis was used to detect the presence of PLN along the purification steps of SERCA2a, starting from pig cardiac SR microsomes. The Western blot analysis showed that purification of SERCA2a removes PLN initially present in the pig cardiac SR microsomes. B) Ca^2+^ concentration-dependent ATPase activity plot of purified SERCA2a. The *V*_max_ calculated from the fitted data is shown inside the plot. Data are reported as the average ± SEM of four biological replicates (*N* = 4).

A conventional concept in the field is that dissociation of the endogenous regulatory SERCA–PLN complex is a requirement for small-molecule activation of the calcium pump (48, 49). However, Western blot analysis showed that SERCA2a purification removes most PLN that is present in the cardiac SR microsomal fraction (Fig. 3A). In this SERCA2a preparations devoid of PLN, we have measured a maximal ATPase activity that falls within the activity range usually obtained using SR microsomes, e.g. 0.46 µmol min^−1^ mg^−1^ for the purified SERCA (Fig. 3B) vs. a *V*_max_ of 0.4–0.6 µmol min^−1^ mg^−1^ typically observed in SR microsomal preparations in our lab. Therefore, the removal of PLN does not increase SERCA2a's turnover rate. Based on this evidence, we propose the hypothesis that CDN1163 does not activate SERCA2a by dissociating PLN from the SERCA2a–PLN complex.

**Fig. 4.**
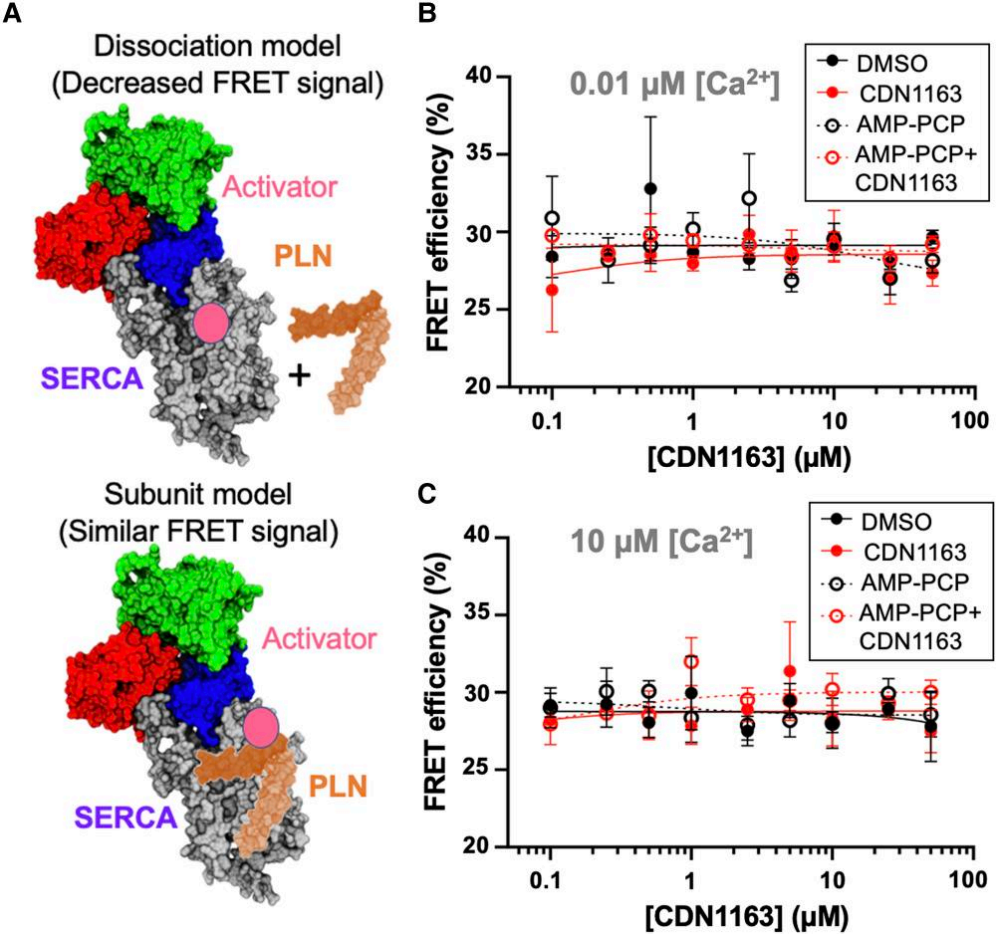
Competitive FRET assays to investigate the effects of CDN1163 on the dissociation of the endogenous SERCA2a–PLN complex. A) Structural representation of the dissociation and subunit models for activation of SERCA2a by CDN1163 tested in this study. Concentration-response curves at increasing CDN1163 concentrations under B) low free Ca^2+^ and C) high free Ca^2+^ conditions. For comparison, we tested the effects of AMP-PCP alone or in the presence of CDN1163. Data are reported as the average ± SEM of four biological replicates (*N* = 4).

We tested this hypothesis using fluorescently labeled SERCA2a and PLN. Here, binding of PLN to SERCA2a produces a FRET signal (measured as the % of FRET efficiency); dissociation of the complex by CDN1163 is shown by the decrease of the FRET signal between SERCA2a and PLN (dissociation model) (50) whereas no changes in the SERCA2a–PLN FRET efficiency suggest that CDN1163 binds to the regulatory complex (subunit model) (50) (Fig. 4A). Activation of SERCA2a requires both Ca^2+^ and ATP, so we performed these studies at low (0.01 µM) and high (10 µM) free Ca^2+^ concentrations, as well as in the presence and absence of the nonhydrolyzable ATP analog AMP-PCP to account for the effects of Ca^2+^ and ATP. At low Ca^2+^, there is no change in the FRET efficiency between SERCA2a and PLN in the presence or absence of AMP-PCP at increasing concentrations of CDN1163 vs. the DMSO vehicle (Fig. 4B). At high Ca^2+^ conditions, the addition of CDN1163 does not influence the FRET efficiency between SERCA2a and PLN in the presence or absence of AMP-PCP vs. the DMSO vehicle (Fig. 4C). A poor fit of the data to a sigmoid curve, i.e. *R*^2^ values <0.18 when fitted to a nonlinear concentration-response regression fit, corroborates the inability of CDN1163 to compete with PLN.

**Fig. 5.**
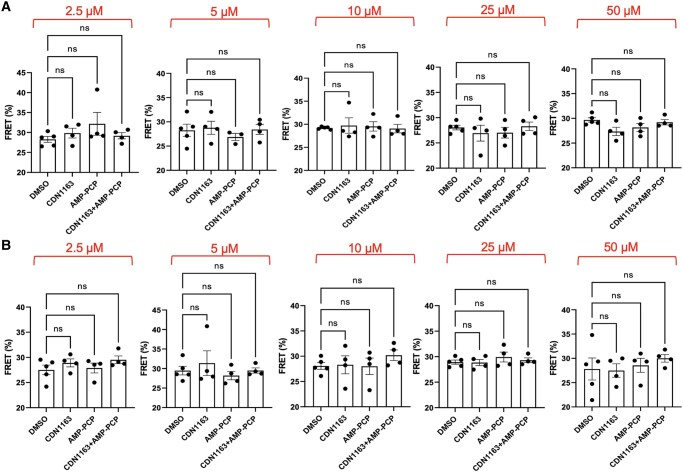
Effects of CDN1163 on the interaction between SERCA2a and PLN. FRET assays were performed at A) low Ca^2+^ conditions (0.01 µM) and B) high Ca^2+^ conditions (10 µM). In all cases, we found that compared to the DMSO vehicle, increasing concentrations of CDN1163 (numbers above the plots) do not significantly change the FRET efficiency between fluorescently labeled SERCA2a and PLN. The addition of the ATP analog AMP-PCP to the SERCA2a- and PLN-containing microsomes does not induce significant changes in the FRET signal vs. the control at both low and high Ca^2+^ conditions. Treatment of the microsomes with a fixed concentration of AMP-PCP and increasing concentrations of CDN1163 does not significantly change the FRET efficiency compared to the vehicle, CDN1163, and AMP-PCP treatment groups. Data is shown as mean ± SEM of four biological replicates (*N* = 4). We used a two-way ANOVA followed by Dunnett's post hoc test to compare treatments (CDN1163, AMP-PCP, and CDN1163+AMP-PCP) against the DMSO vehicle; *ns* = not significant.

Our primary aim was to assess the concentration-response competition between CD1163 and PLN for binding to SERCA2a, and our analysis did not show a sigmoid relationship between CDN1163 concentrations and the FRET signal between SERCA2a and PLN. Nonetheless, there may be small, yet significant differences in the SERCA2a–PLN FRET signal at different concentrations of CDN1163. To test this, we performed a two-way ANOVA to establish changes in the FRET signal in response to CDN1163, and the potential effects of Ca^2+^ or ATP on the competition between CDN1163 and PLN compared to the DMSO vehicle. The statistical analysis showed that the FRET efficiency between SERCA2a and PLN does not significantly change in the presence of CDN1163 at low (Fig. 5A) or high Ca^2+^ (Fig. 5B) at all concentrations of the compound, including those near the half activation (i.e. 2.5 µM, Fig. 5A) and saturating (i.e. 10 and 50 µM, Fig. 5A). Addition of AMP-PCP does not significantly change the SERCA2a–PLN FRET at functionally relevant concentrations of the compound. These findings reveal that CDN1163 does not activate SERCA2a by displacing PLN from the regulatory SERCA2a–PLN complex.

### Effects of ATP on the structural dynamics of SERCA2a

ATP's traditional role has been confined as a source of energy to power the Ca^2+^-transporting activity of SERCA, but it has also been suggested that the nucleotide-binding site plays a role in the so-called “distal allostery” of SERCA (51). Therefore, we used TCSPC imaging to investigate whether ATP's role extends beyond a substrate, also acting as a modulator of SERCA2a structural dynamics. For these experiments, we use the nonhydrolyzable ATP analog AMP-PCP to eliminate ATP hydrolysis as a confounding, thus, allowing us to isolate the effects of ATP binding on the structural dynamics of the pump. Since our focus is on the structural shifts that correlate with the formation of catalytically competent populations, we, therefore, represent the structural shifts as the percent in the closed population of the headpiece.

**Fig. 6.**
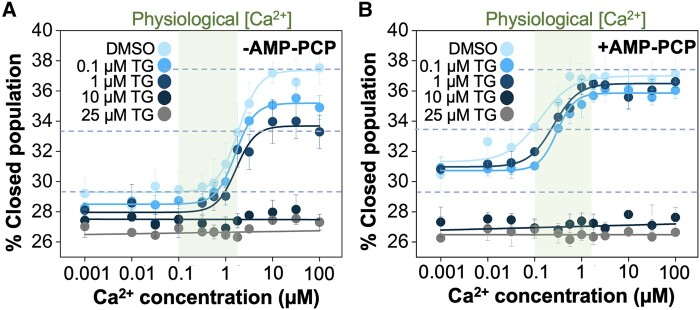
Structural changes of SERCA2a in response to the ATP analog AMP-PCP and the inhibitor TG. Twelve-point Ca^2+^ concentration-response curves were obtained in the A) absence and B) presence of AMP-PCP at four concentrations of TG. In both cases, we include a DMSO vehicle as a control. The structural change in response to ligands is shown as the % of the closed population of the headpiece. The dashed lines show the lower, midpoint, and upper boundaries of the concentration-response curve of SERCA2a in the absence of AMP-PCP and TG. The green shaded area highlights the range of Ca^2+^ concentrations at which SERCA2a is operational in cardiac cells. Data are reported as average ±SEM of four biological replicates (*N* = 4).

We first determined the structural shifts of SERCA in the presence and absence of AMP-PCP; in addition, we tested different concentrations of TG, a selective calcium pump inhibitor (52), as a control. In the absence of both AMP-PCP and TG, SERCA2a undergoes a transition toward a higher population of the closed state in a Ca^2+^ concentration-dependent manner (Fig. 6A). This Ca^2+^-dependent response of the two-color SERCA2a agrees with previous studies showing that increasing concentrations of Ca^2+^ are sufficient to induce a redistribution of structural populations in the pump (32). We found that the population of the closed state goes from ∼29.5% at nM [Ca^2+^] concentrations to ∼37.5% at saturating µM of [Ca^2+^]. The addition of 0.1 and 1 µM TG in the absence of AMP-PCP decreases the fraction of the closed population both at nM and µM Ca^2+^ concentrations (Fig. 6A); however, this effect is more pronounced at µM Ca^2+^ concentrations, where TG significantly decreases the closed population both at 0.1 µM (*P* = 0.0001) and 1 µM (*P* = 0.0008) of the inhibitor. Yet, TG has no significant effect on the affinity for Ca^2+^, e.g. K_Ca_ values of 1.85 ± 0.19 µM and K_Ca_ = 1.66 ± 0.34 µM in the absence and the presence of 1 µM TG, respectively. The Ca^2+^-dependent increase in the closed population is completely blunted at high (>10 µM) concentrations of TG in the absence of AMP-PCP (Fig. 6A). This agrees with previous studies have shown that Ca^2+^ binding to the pump modulates the structural dynamics of the cytosolic headpiece (32, 37, 51, 53–55).

SERCA2a also undergoes a structural transition toward a higher population of the closed state in a Ca^2+^ concentration-dependent manner upon the addition of AMP-PCP (Fig. 6B). Addition of AMP-PCP and TG (0.1 and 1 µM) does not decrease the fraction of the closed population at µM [Ca^2+^]. Interestingly, we found that AMP-PCP substantially increases the fraction of the closed conformation population at nM Ca^2+^ concentrations both in the presence and absence of TG (Fig. 6B). The functional consequences of this effect are illustrated in Fig. 6: In the absence of AMP-PCP, Ca^2+^-dependent SERCA2a activation curve reaches a midpoint and a plateau at Ca^2+^ concentrations of ∼2 and ∼10 µM, respectively. Upon addition of AMP-PCP, the curves reach a midpoint and a plateau at Ca^2+^ concentrations range of 0.4–0.5 and 1–2 µM, respectively. Remarkably, the increase in the fraction of catalytically competent structures predominantly occurs within the physiological Ca^2+^ concentrations at which SERCA operates (Fig. 6A and B; shaded area) (56–58).

In the presence of AMP-PCP, TG concentrations of 0.1 and 1 µM significantly (*P* < 0.01) decrease the affinity of SERCA2a for Ca^2+^, showing that AMP-PCP partially reverses the inhibition of Ca^2+^-induced structural transitions in the pump. However, the structural transitions induced by AMP-PCP and Ca^2+^ were completely inhibited at high (>10 µM) concentrations of TG both in the presence and absence of AMP-PCP (Fig. 6A and B). These findings correlate with studies showing that TG inhibits both calcium loading (59) and the formation of catalytically competent SERCA structures (32). Yet, our findings show more: AMP-PCP is a direct effector of SERCA2a, inducing structural changes in the headpiece of the pump at nonsaturating nanomolar Ca^2+^ concentrations, and partially reversing the inhibitory effects of TG on the population shifts required for activation of SERCA2a. We note that TG fully inhibits SERCA2a's structural transitions at concentrations higher than 10 µM, but does not shift the fraction of the closed population below 26% (Fig. 6A). We also found that AMP-PCP does not shift the fraction of the closed population above 37% (Fig. 6B). Therefore, these values represent the lower and upper limits between population fractions occupied during Ca^2+^-and AMP-PCP-dependent structural transitions of the pump.

### CDN1163 enhances the ATP-mediated modulatory effects to increase the population of catalytically competent SERCA2a structures

We showed ATP is a modulator of SERCA2a's structural dynamics, so we tested whether the activator CDN1163 operates through a similar structural mechanism. We first tested whether CDN1163 activates the two-color SERCA2a construct used for the TCSPC imaging experiments. ATPase activity assays using endoplasmic reticulum (ER) microsomes from HEK-293 cells infected with adenovirus showed that the two-color SERCA2a has a *V*_max_ of 0.058 ± 0.001 µmol min^−1^ mg^−1^. Incubation of ER microsomes with CDN1163 (5–10 µM) showed that this activator increases the activity of the two-color SERCA2a by 4% relative to the vehicle control. The effect of CDN1163 on the two-color SERCA, while significant (*P* < 0.05) is lower than that observed for the cardiac SR preparation. However, this finding is not unexpected because the ATPase assay is dependent on the protein concentration, and cardiac tissue yields substantially more SERCA2a than the HEK-293 cells. The lower protein yield is consistent with a *V*_max_ of the two-color SERCA2a that is 6–10 times lower than that of the protein isolated from cardiac tissue. Yet, the ATPase activity assays show that the two-color SERCA is catalytically active and that CDN1163 stimulates the activity of this construct.

**Fig. 7.**
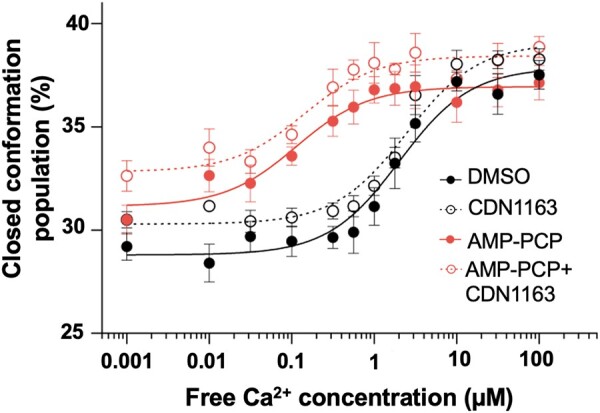
Structural changes of SERCA2a in response to CDN1163. Twelve-point Ca^2+^ concentration-response curves were obtained in the presence of DMSO vehicle, CDN1163, AMP-PCP, and CDN1163 + AMP-PCP. The structural change in response to ligands is shown as the % of the closed population of the headpiece. Data are reported as average ±SEM of four biological replicates (*N* = 4).

We next performed TCSPC imaging to show that in the absence of CDN1163 and at low Ca^2+^ (i.e. [Ca^2+^] < 0.1 µM), ∼28% of the SERCA2a populations are in the closed structure. Increasing Ca^2+^ concentrations shift the population of the closed structure of SERCA2a to ∼37% (Fig. 6). Incubation of SERCA2a with either CDN1163 or AMP-PCP induces a shift in the population of the headpiece toward a closed structure, although the effect is more pronounced in the presence of AMP-PCP (Fig. 7). Interestingly, incubation of SERCA2a with both CDN1163 and AMP-PCP further shifts the population of the headpiece toward a closed structural state that is comparable to that of SERCA2a alone at saturating Ca^2+^ conditions (Fig. 7). The cooperative effects of CDN1163 and AMP-PCP are more prominent at Ca^2+^ concentrations between 0.1 and 2 µM, although this shift in the structural populations was not observed at [Ca^2+^] > 2 µM (Fig. 7). These findings suggest that CDN1163 enhances the Ca^2+^-dependent modulatory effect of ATP to increase the population of catalytically competent SERCA2a structures. We tested this hypothesis by statistical analysis of the changes in the % of the closed headpiece SERCA2a in response to CDN1163, AMP-PCP, and CDN1163/AMP-PCP at 12 Ca^2+^ concentrations.

**Fig. 8.**
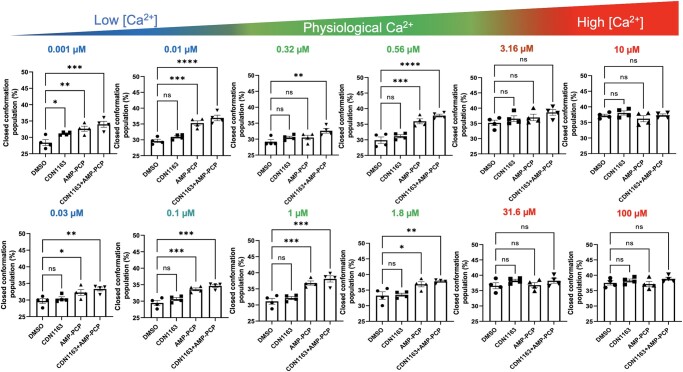
Effects of CDN1163, AMP-PCP, and CDN1163/AMP-PCP on the structural populations of SERCA2a. TCSPC assays were performed at increasing Ca^2+^ conditions from 0.001 µM to 100 µM. Intracellular free Ca^2+^ concentration in cardiac cells fluctuates in the range of 1–2.65 µM and 0.1–0.16 µM during contraction and relaxation of the heart. Therefore, these values represent the upper and lower limits of the range of physiological Ca^2+^ concentrations at which SERCA2a operates. The full range of Ca^2+^ concentrations used in this study is shown above the plots. In all cases, we used AMP-PCP and CDN1163 concentrations of 0.5 and 10 µM, respectively. Data is shown as mean ± SEM of four biological replicates (*N* = 4). We used a two-way ANOVA followed by the Dunnett's post hoc test to compare treatments (CDN1163, AMP-PCP, and CDN1163/AMP-PCP) against the DMSO vehicle. *P < 0.05; **P < 0.01; ***P < 0.001; **** *P* < 0.0001.

During cardiac contraction, the intracellular free Ca^2+^ concentration in cardiac cells increases to 1–2.65 µM, allowing interaction between the contractile elements (56–58). Relaxation occurs following a decrease in free Ca^2+^ concentrations to 0.1–0.16 µM, causing dissociation of the contractile elements (56–58). Therefore, these values represent the upper and lower limits of the range of physiological Ca^2+^ concentrations at which SERCA2a is operational in cardiac cells. The statistical analysis showed that compared to the DMSO vehicle, CDN1163 alone does not have a significant effect on the headpiece population at most Ca^2+^ concentrations tested here (Fig. 8). CDN1163 alone significantly shifts the headpiece populations toward a closed conformation (*P* < 0.05, Fig. 8) at a Ca^2+^ concentration outside the physiological window ([Ca^2+^] = 0.001 µM). Compared to the DMSO vehicle, AMP-PCP induces a significant shift in SERCA2a's headpiece toward a closed population at Ca^2+^ concentrations 0.001, 0.01, 0.03, 0.1 0.56, 1, and 1.8 µM (Fig. 8). The effects of AMP-PCP on SERCA2a agree with previous studies showing that the binding of ATP induces closure of the pump's headpiece (51). This effect is not significant at a [Ca^2+^] = 0.32 µM, which falls within the physiological window of Ca^2+^ concentrations (Fig. 8). AMP-PCP also does not have a significant effect on the headpiece populations at Ca^2+^ concentrations at high Ca^2+^ conditions outside the physiological window (Fig. 8). The most significant effect on the population shift occurs in the presence of both CDN1163 and AMP-PCP at most Ca^2+^ concentrations below 2 µM, e.g. *P* < 0.05 with AMP-PCP vs. *P* < 0.01 with AMP-PCP and CDN1163 at a free Ca^2+^ concentration of 1.8 µM (Fig. 8). The treatment of microsomes with both AMP-PCP and CDN1163 does not have a significant effect on the headpiece populations at high (>1.8 µM) Ca^2+^ concentrations that fall outside physiologically relevant Ca^2+^ concentrations (Fig. 8).

### Effects of CDN1163 on the affinity of SERCA2a for Ca^2+^ ions

**Fig. 9.**
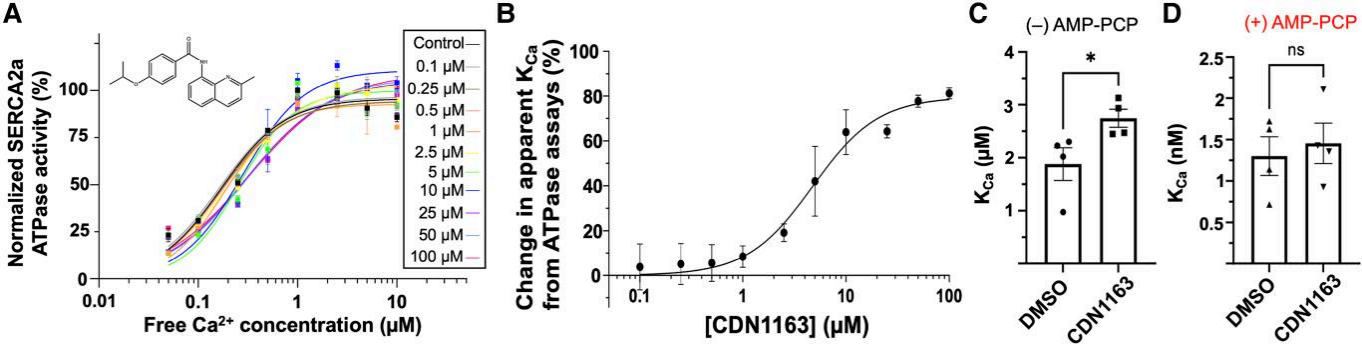
Effects of CDN1163 on SERCA2a's affinity for Ca^2+^ ions. A) Eight-point Ca^2+^ concentration-response of SERCA2a ATPase activity curves obtained at 10 concentrations of CDN1163 (0.1 to 100 µM). The activity at each concentration of CDN1163 was normalized relative to the control at [Ca^2+^] = 1 µM because this is the point representing the maximal activity of the pump in our assays. B) Changes in apparent Ca^2+^ affinity (K_Ca_) induced by increasing concentrations of CDN1163; the apparent affinity is expressed as the %change relative to the vehicle control. Data are reported as average ± SEM of three biological replicates (*N* = 3). To eliminate the confounding effects of ATPase hydrolysis, we used TCSPC imaging to measure the effects of 10 µM CDN1163 on SERCA2a's K_Ca_ in the C) absence and D) presence of the ATP analog AMP-PCP. Data is shown as mean ± SEM of four biological replicates (*N* = 4). Significance compared to the DMSO vehicle was determined using a two-tailed Mann–Whitney *U* test; *P < 0.05.

We showed that CDN1163 potentiates the effects of AMP-PCP to produce a population shift required for the activation of the pump. Besides these structural changes, it may be possible that CDN1163 activates SERCA2a by increasing its affinity for Ca^2+^. We tested this mechanism first by analyzing the [Ca^2+^]-dependent ATPase activity curves of SERCA2a at compound concentrations between 0.1–100 µM. We found that at concentrations of CDN1163 equal to or less than 1 µM, the compound does not affect the Ca^2+^ affinity for SERCA, illustrating the fact that concentration-response curves do not shift along the *x*-axis compared to the control (Fig. 9A). However, at concentrations of CDN1163 equal or higher than 2.5 µM, the concentration-response curves shift to the right along the *x*-axis.

The ATPase activity curves suggest that while CDN1163 increases the maximal activity of SERCA2a (i.e. turnover rate) (Fig. 9A), it also decreases the apparent SERCA2a's affinity for Ca^2+^ (Fig. 9B). A limitation of the ATPase activation assays is that the signal depends on hydrolysis of ATP, and therefore, it is impossible to separate the effects of the activator alone or in combination with nucleotide substrate on SERCA2a's affinity for Ca^2+^. We overcome this limitation by calculating SERCA2a's affinity for Ca^2+^ directly from the TCSPC imaging curves (Fig. 7). The advantage of this approach is that we eliminate the confounding effects of ATPase hydrolysis. We determined the effect of CDN1163 on SERCA's K_Ca_ at a concentration of 10 µM because it exerts the maximal stimulatory effect in the ATPase activity assays (Fig. 9A). The calculated K_Ca_ of SERCA2a in the DMSO vehicle and the absence of AMP-PCP is 1.9 ± 0.3 µM, and treatment of the microsomes with CDN1163 significantly increases the K_Ca_ of the pump to a value of 2.7 ± 0.2 µM (*P* = 0.0286, Fig. 9C). In the presence of AMP-PCP, the K_Ca_ of SERCA2a is 1.3 ± 0.2 nM and addition of 10 µM CDN1163 decreases the pump's affinity for Ca^2+^, with a mean K_Ca_ value 1.5 ± 0.2 nM (Fig. 9D). These findings agree with the ATPase activity assays and indicate that CDN1163 decreases the SERCA2a's affinity for Ca^2+^. However, SERCA2a activation requires the presence of nucleotide, and the effect of CDN1163 on K_Ca_ is not significant in the presence of AMP-PCP (*P* = 0.8857, Fig. 9D). Collectively, the ATPase assays and TCSPC imaging experiments indicate that CDN1163 does not increase the affinity of SERCA2a for Ca^2+^ ions at functionally relevant conditions of the pump.

## Discussion

We developed a TCSPC imaging approach to determine the mechanisms for the activation of SERCA2a. This approach, enabled by the recent development of improved fluorescence proteins and hybrid detectors for TCSPC, allows us to detect the individual and combined effects of Ca^2+^, nucleotide substrate, and small-molecule modulators on the structural dynamics of SERCA2a. TCSPC allows the measurement of changes in the fluorescence lifetimes for complex mixtures of fluorescent species. The benefits of this method are a strong statistical background and independence of this method on the concentration of the fluorescence species. The sensitivity of this method is two orders of magnitude more sensitive than methods using concentration-dependent FRET measurements (e.g. acceptor sensitization FRET) (32, 37). Changes in FRET are correlated with changes in the amplitude-weighted average lifetime and together with multi-exponential fitting of the whole range of Ca^2+^ concentration enable us to monitor changes in two major fluorescent species. The powerful combination of TCSPC imaging and component analysis yielded structural insights into mechanisms for SERCA2a activation by a small-molecule modulator in unprecedented detail.

We first used ATPase activation assays to screen for SERCA2a effectors that activate SERCA in a concentration-dependent manner. This is important because there are small-molecule probes that exhibit a “bell” or “U-shaped” behavior, which indicates compound-mediated assay interference (46), and therefore, renders these molecules unsuitable as molecular probes. Among the SERCA effectors reported in the literature, only CDN1163 acts as a direct activator of SERCA2a, increasing its activity sigmoidally, and with an effect on *V*_max_ kept at higher concentrations of the compound. These findings agree with a solid-supported membrane biosensing approach showing that CDN1163 enhances SERCA-mediated Ca^2+^ translocation at compound concentrations that are similar to those in this study (40). An important question we ask is whether the ∼12% activation of SERCA2a ATPase activity is functionally significant at the cellular level. We have previously used optical mapping experiments to show that CDN1163 significantly increases Ca^2+^ dynamics in cardiac cells (47). These functional effects are comparable to those induced by the adrenergic agonist isoproterenol, which promotes SERCA2a activation via PLN phosphorylation (14, 60, 61). These studies validate CDN1163 as a probe to systematically characterize the mechanisms for small-molecule activation of SERCA2a.

PLN inhibits SERCA2a by binding to a large pocket in the transmembrane domain of the pump (62–67). This interaction populates a Ca^2+^ ion-free intermediate that serves as a kinetic trap that decreases SERCA's apparent affinity for Ca^2+^ and depresses the structural transitions necessary for Ca^2+^-dependent activation of the pump (68–70). Therefore, a conventional concept in the field is that dissociation of the SERCA2a–PLN complex is a requirement for SERCA2a reactivation (48). Therefore, we tested whether CDN1163 stimulates the ATPase activity of SERCA2a by displacing PLN from the endogenous SERCA2a–PLN complex. We found CDN1163 does not dissociate the complex even at saturating concentrations of the compound. PLN is thought to interact with Ca^2+^-free forms of SERCA and partially dissociate from the pump at high Ca^2+^ concentrations (71, 72). However, CDN1163 has no effect on the FRET signal between SERCA2a and PLN at saturating Ca^2+^ conditions (i.e. 10 µM). Previous studies have also suggested that PLN and ATP stabilize a functional SERCA–PLN–ATP state that protects the pump against the binding of inhibitors (73). We found that in the presence of the ATP analog AMP-PCP at either low or high Ca^2+^ conditions, CDN1163 has no effect on the FRET signal between SERCA2a and PLN, showing that CDN1163 does not compete with PLN and has no effect on the PLN/ATP-bound state of the pump. This evidence shows that CDN1163 does not dissociate the SERCA2a–PLN complex, challenging the current paradigm proposing that endogenous PLN needs to be displaced from SERCA to activate the pump (48, 49).

We take advantage of the robustness of the TCSPC imaging method, which has over four orders of magnitude difference between signal and noise. We complement these experiments with a global analysis of lifetimes for over 200 fluorescence decays and attribute specific lifetimes to structural states of SERCA2a. The combination of TCSPC and global analysis allows us to resolve the structural states of SERCA2a and determine how these states are redistributed in response to ligands. An important advantage of this approach is that we examine the effects of Ca^2+^, ATP, and small-molecule activators alone or in combination. The use of complementary TCSPC imaging and global analysis approach resolved two primary structural populations of SERCA2a: a catalytically inactive open state, and a closed state that brings together the structural elements required for the formation of a compact headpiece. Indeed, previous studies have shown that the active, disinhibited conformation of SERCA samples is more compact, well-ordered conformations that are catalytically competent (24, 51, 74).

We used this combined approach to first probe the effects of ATP on the dynamics of SERCA2a. We found that in general, the Ca^2+^-dependent response resolved by the TCSCP imaging in the presence of AMP-PCP is strikingly similar to that observed in ATPase activity assays, where the midpoint of the curve is observed at Ca^2+^ concentrations of 0.45–0.5 µM, and the *V*_max_ is usually reached at Ca^2+^ concentrations of 1–2.5 µM (29). We note that while TCSPC imagining measures structural changes and ATPase assays measure SERCA2a's catalytic turnover, the correlation between the two techniques indicates that our approach captures the structural transitions associated with SERCA2a activation. These findings also suggest that ATP primes SERCA2a for activation by increasing the fraction of catalytically competent structures of the pump at physiologically relevant Ca^2+^ concentrations in cardiac cells. ATP and AMP-PCP form virtually identical bound complexes with the pump (75), so these findings indicate that ATP is both a substrate and a modulator of SERCA2a dynamics. We then asked whether CDN1163 activates SERCA by a mechanism that is similar to that of ATP. We found that, unlike ATP, CDN1163 alone has no significant effects on the Ca^2+^-dependent shifts in the structural populations of SERCA2a, and it does not modulate the pump's affinity for Ca^2+^ ions at functionally relevant conditions. Instead, we found that the combined effect of CDN1163 and AMP-PCP induces a structural shift of the headpiece population that is comparable to that at saturating Ca^2+^ conditions. This population shift occurs only within or below physiological Ca^2+^ concentrations (56–58). Based on these findings, we propose that CDN1163 activates SERCA2a by enhancing the ATP-mediated modulatory effects to increase the population of structures that are primed for activation.

Crystallography, spectroscopy, and computational studies have shown that activation and inhibition of SERCA correlate with the shifts between open and closed structural populations of the cytosolic headpiece, the domain of the pump that contains the catalytic elements of the pump (32). Specifically, activation of the pump occurs through a population shift toward a closed structure of the headpiece, whereas inhibition by small molecules (e.g. TG) and endogenous PLN occurs through a shift in the headpiece populations toward an open state (32, 37, 74). While our study correlates well with this mechanism, it shows for the first time how these structural changes occur in response to ligands, nucleotide substrate, and an allosteric modulator quantitatively. This is important because TCSPC experiments at all conditions show that SERCA2a's headpiece undergoes a maximal ∼10% shift in the equilibrium toward the closed state in activating conditions. Relatively small shifts in structural populations are a common theme in the allosteric modulation of sarcomeric proteins. For example, allosteric activation of the smooth muscle myosin induced by phosphorylation of its regulatory light chain occurs with a ∼20% shift in the structural populations within its phosphorylation domain (76).

In conclusion, in this work we introduce TCSPC imaging and global analysis of fluorescent lifetimes, a powerful approach that makes possible the direct analysis of the structural mechanisms underlying allosteric modulation of proteins. A key feature of this combined approach is the resolution of protein structural states and population shifts in response to ligands, substrates, and small molecules. This technical advantage allowed us to monitor the structural mechanisms for activation of the cardiac calcium pump SERCA2a by CDN1163, a validated small-molecule allosteric modulator. The significance of this space-time resolution is 3-fold. First, the experiments directly showed that CDN1163 does not compete for binding with SERCA2a's endogenous regulator PLN and that this effect is independent of Ca^2+^ and ATP. Second, the method allowed us to directly measure the response of SERCA2a to ligands and show that CDN1163 does not activate SERCA2a by modulating the pump's affinity for Ca^2+^. Finally, we showed that CDN1163 and ATP act synergistically to populate SERCA2a structures that are primed for ATP utilization and SERCA2a's phosphorylation. In summary, this study provides novel insights into the synergy between SERCA2a's substrate and a synthetic allosteric modulator to activate a clinically important target in the heart.

## Data Availability

All data are available in the main text.

## References

[1] Han B , Salituro FG, Blanco MJ. 2020. Impact of allosteric modulation in drug discovery: innovation in emerging chemical modalities. ACS Med Chem Lett. 11(10):1810–1819. 33062158 10.1021/acsmedchemlett.9b00655 PMC7549105

[2] Lu S , Li S, Zhang J. 2014. Harnessing allostery: a novel approach to drug discovery. Med Res Rev. 34(6):1242–1285. 24827416 10.1002/med.21317

[3] Motlagh HN , Wrabl JO, Li J, Hilser VJ. 2014. The ensemble nature of allostery. Nature. 508(7496):331–339. 24740064 10.1038/nature13001 PMC4224315

[4] Nussinov R , Tsai CJ. 2013. Allostery in disease and in drug discovery. Cell. 153(2):293–305. 23582321 10.1016/j.cell.2013.03.034

[5] Reynolds KA , McLaughlin RN, Ranganathan R. 2011. Hot spots for allosteric regulation on protein surfaces. Cell. 147(7):1564–1575. 22196731 10.1016/j.cell.2011.10.049 PMC3414429

[6] Guarnera E , Berezovsky IN. 2020. Allosteric drugs and mutations: chances, challenges, and necessity. Curr Opin Struct Biol. 62:149–157. 32062398 10.1016/j.sbi.2020.01.010

[7] Periasamy M , Huke S. 2001. SERCA pump level is a critical determinant of Ca(2+)homeostasis and cardiac contractility. J Mol Cell Cardiol. 33(6):1053–1063. 11444913 10.1006/jmcc.2001.1366

[8] Yu X , Carroll S, Rigaud JL, Inesi G. 1993. H+ countertransport and electrogenicity of the sarcoplasmic reticulum Ca2+ pump in reconstituted proteoliposomes. Biophys J. 64(4):1232–1242. 8388268 10.1016/S0006-3495(93)81489-9 PMC1262440

[9] Zafar S , Hussain A, Liu Y, Lewis D, Inesi G. 2008. Specificity of ligand binding to transport sites: Ca2+ binding to the Ca2+ transport ATPase and its dependence on H+ and Mg2+. Arch Biochem Biophys. 476(1):87–94. 18485884 10.1016/j.abb.2008.04.035 PMC2756220

[10] Cantilina T , Sagara Y, Inesi G, Jones LR. 1993. Comparative studies of cardiac and skeletal sarcoplasmic reticulum ATPases. Effect of a phospholamban antibody on enzyme activation by Ca2+. J Biol Chem. 268(23):17018–17025. 8349590

[11] Sahoo SK , Shaikh SA, Sopariwala DH, Bal NC, Periasamy M. 2013. Sarcolipin protein interaction with sarco(endo)plasmic reticulum Ca2+ ATPase (SERCA) is distinct from phospholamban protein, and only sarcolipin can promote uncoupling of the SERCA pump. J Biol Chem. 288(10):6881–6889. 23341466 10.1074/jbc.M112.436915 PMC3591597

[12] Chien KR , Ross J, Jr., Hoshijima M. 2003. Calcium and heart failure: the cycle game. Nat Med. 9(5):508–509. 12724757 10.1038/nm0503-508

[13] Simmerman HK , Jones LR. 1998. Phospholamban: protein structure, mechanism of action, and role in cardiac function. Physiol Rev. 78(4):921–947. 9790566 10.1152/physrev.1998.78.4.921

[14] Simmerman HK , Collins JH, Theibert JL, Wegener AD, Jones LR. 1986. Sequence analysis of phospholamban. Identification of phosphorylation sites and two major structural domains. J Biol Chem. 261(28):13333–13341. 3759968

[15] Ablorh NA , et al 2014. Synthetic phosphopeptides enable quantitation of the content and function of the four phosphorylation states of phospholamban in cardiac muscle. J Biol Chem. 289(42):29397–29405. 25190804 10.1074/jbc.M114.556621 PMC4200288

[16] Mattiazzi A , Mundina-Weilenmann C, Guoxiang C, Vittone L, Kranias E. 2005. Role of phospholamban phosphorylation on Thr17 in cardiac physiological and pathological conditions. Cardiovasc Res. 68(3):366–375. 16226237 10.1016/j.cardiores.2005.08.010

[17] Minamisawa S , et al 1999. Chronic phospholamban-sarcoplasmic reticulum calcium ATPase interaction is the critical calcium cycling defect in dilated cardiomyopathy. Cell. 99(3):313–322. 10555147 10.1016/s0092-8674(00)81662-1

[18] Baker JE , LaConte LE, Thomas DD, Brust-Mascher I. 2000. Muscle chemistry and force. Biophys J. 79(3):1687–1688. 11041630 10.1016/S0006-3495(00)76419-8 PMC1301061

[19] Nesmelov YE , Surek JT, Thomas DD. 2001. Enhanced EPR sensitivity from a ferroelectric cavity insert. J Magn Reson. 153(1):7–14. 11700076 10.1006/jmre.2001.2415

[20] Meyer M , et al 2004. A recombinant antibody increases cardiac contractility by mimicking phospholamban phosphorylation. Faseb J. 18(11):1312–1314. 15180962 10.1096/fj.03-1231fje

[21] Hoshijima M , et al 2002. Chronic suppression of heart-failure progression by a pseudophosphorylated mutant of phospholamban via in vivo cardiac rAAV gene delivery. Nat Med. 8(8):864–871. 12134142 10.1038/nm739

[22] Kaye DM , Hoshijima M, Chien KR. 2008. Reversing advanced heart failure by targeting Ca2+ cycling. Annu Rev Med. 59:13–28. 18186701 10.1146/annurev.med.59.052407.103237

[23] Hajjar RJ , et al 2008. Design of a phase 1/2 trial of intracoronary administration of AAV1/SERCA2a in patients with heart failure. J Card Fail. 14(5):355–367. 18514926 10.1016/j.cardfail.2008.02.005

[24] Aguayo-Ortiz R , Espinoza-Fonseca LM. 2020. Linking biochemical and structural states of SERCA: achievements, challenges, and new opportunities. Int J Mol Sci. 21(11):4146. 32532023 10.3390/ijms21114146 PMC7313052

[25] Dyla M , Basse Hansen S, Nissen P, Kjaergaard M. 2019. Structural dynamics of P-type ATPase ion pumps. Biochem Soc Trans. 47(5):1247–1257. 31671180 10.1042/BST20190124

[26] Dyla M , Kjaergaard M, Poulsen H, Nissen P. 2020. Structure and mechanism of P-type ATPase ion pumps. Annu Rev Biochem. 89:583–603. 31874046 10.1146/annurev-biochem-010611-112801

[27] Primeau JO , Armanious GP, Fisher ME, Young HS. 2018. The SarcoEndoplasmic reticulum calcium ATPase. Subcell Biochem. 87:229–258. 29464562 10.1007/978-981-10-7757-9_8

[28] Rathod N , et al 2021. Nothing regular about the regulins: distinct functional properties of SERCA transmembrane peptide regulatory subunits. Int J Mol Sci. 22(16):8891. 34445594 10.3390/ijms22168891 PMC8396278

[29] Sitsel A , et al 2019. Structures of the heart specific SERCA2a Ca(2+)-ATPase. EMBO J. 38(5):e100020. 30777856 10.15252/embj.2018100020 PMC6396164

[30] Cruz-Cortes C , et al 2023. A novel machine learning-based screening identifies statins as inhibitors of the calcium pump SERCA. J Biol Chem. 299(5):104681. 37030504 10.1016/j.jbc.2023.104681 PMC10193016

[31] Schoenmakers TJ , Visser GJ, Flik G, Theuvenet AP. 1992. CHELATOR: an improved method for computing metal ion concentrations in physiological solutions. Biotechniques. 12(6):870–874, 876-9. 1642895

[32] Raguimova ON , et al 2018. Redistribution of SERCA calcium pump conformers during intracellular calcium signaling. J Biol Chem. 293(28):10843–10856. 29764938 10.1074/jbc.RA118.002472 PMC6052202

[33] Laviv T , et al 2016. Simultaneous dual-color fluorescence lifetime imaging with novel red-shifted fluorescent proteins. Nat Methods. 13(12):989–992. 27798609 10.1038/nmeth.4046 PMC5322478

[34] Bajar BT , et al 2016. Fluorescent indicators for simultaneous reporting of all four cell cycle phases. Nat Methods. 13(12):993–996. 27798610 10.1038/nmeth.4045 PMC5548384

[35] Bovo E , et al 2020. Dimerization of SERCA2a enhances transport rate and improves energetic efficiency in living cells. Biophys J. 119(7):1456–1465. 32946770 10.1016/j.bpj.2020.08.025 PMC7567987

[36] Schaaf TM , et al 2018. Red-Shifted FRET biosensors for high-throughput fluorescence lifetime screening. Biosensors (Basel). 8(4):99. 30352972 10.3390/bios8040099 PMC6315989

[37] Raguimova ON , Aguayo-Ortiz R, Robia SL, Espinoza-Fonseca LM. 2020. Dynamics-driven allostery underlies Ca(2+)-mediated release of SERCA inhibition by phospholamban. Biophys J. 119(9):1917–1926. 33069270 10.1016/j.bpj.2020.09.014 PMC7677127

[38] Ceholski DK , Trieber CA, Young HS. 2012. Hydrophobic imbalance in the cytoplasmic domain of phospholamban is a determinant for lethal dilated cardiomyopathy. J Biol Chem. 287(20):16521–16529. 22427649 10.1074/jbc.M112.360859 PMC3351288

[39] Seflova J , et al 2022. Fluorescence lifetime imaging microscopy reveals sodium pump dimers in live cells. J Biol Chem. 298(5):101865. 35339486 10.1016/j.jbc.2022.101865 PMC9048134

[40] Sordi G , Goti A, Young HS, Palchetti I, Tadini-Buoninsegni F. 2021. Stimulation of Ca(2+) -ATPase transport activity by a small-molecule drug. ChemMedChem. 16(21):3293–3299. 34297466 10.1002/cmdc.202100350 PMC8571031

[41] Cornea RL , et al 2013. High-throughput FRET assay yields allosteric SERCA activators. J Biomol Screen. 18(1):97–107. 22923787 10.1177/1087057112456878 PMC3721969

[42] Rocchetti M , et al 2008. Modulation of sarcoplasmic reticulum function by PST2744 [istaroxime; (E, Z)-3-((2-aminoethoxy)imino) androstane-6,17-dione hydrochloride)] in a pressure-overload heart failure model. J Pharmacol Exp Ther. 326(3):957–965. 18539651 10.1124/jpet.108.138701

[43] Stroik DR , et al 2018. Targeting protein-protein interactions for therapeutic discovery via FRET-based high-throughput screening in living cells. Sci Rep. 8(1):12560. 30135432 10.1038/s41598-018-29685-z PMC6105598

[44] Ferrandi M , et al 2013. Istaroxime stimulates SERCA2a and accelerates calcium cycling in heart failure by relieving phospholamban inhibition. Br J Pharmacol. 169(8):1849–1861. 23763364 10.1111/bph.12278 PMC3753840

[45] Micheletti R , et al 2002. Pharmacological profile of the novel inotropic agent (E, Z)-3-((2-aminoethoxy)imino)androstane-6,17-dione hydrochloride (PST2744). J Pharmacol Exp Ther. 303(2):592–600. 12388640 10.1124/jpet.102.038331

[46] Owen SC , et al 2014. Colloidal drug formulations can explain “bell-shaped” concentration-response curves. ACS Chem Biol. 9(3):777–784. 24397822 10.1021/cb4007584 PMC3985758

[47] Aguayo-Ortiz R , et al 2021. A multiscale approach for bridging the gap between potency, efficacy, and safety of small molecules directed at membrane proteins. Sci Rep. 11(1):16580. 34400719 10.1038/s41598-021-96217-7 PMC8368179

[48] Stroik DR , et al 2020. Viral expression of a SERCA2a-activating PLB mutant improves calcium cycling and synchronicity in dilated cardiomyopathic hiPSC-CMs. J Mol Cell Cardiol. 138:59–65. 31751570 10.1016/j.yjmcc.2019.11.147 PMC7035975

[49] Cleary SR , et al 2022. Inhibitory and stimulatory micropeptides preferentially bind to different conformations of the cardiac calcium pump. J Biol Chem. 298(7):102060. 35605666 10.1016/j.jbc.2022.102060 PMC9218510

[50] Dong X , Thomas DD. 2014. Time-resolved FRET reveals the structural mechanism of SERCA-PLB regulation. Biochem Biophys Res Commun. 449(2):196–201. 24813991 10.1016/j.bbrc.2014.04.166 PMC4054823

[51] Autry JM , Rubin JE, Svensson B, Li J, Thomas DD. 2012. Nucleotide activation of the ca-ATPase. J Biol Chem. 287(46):39070–39082. 22977248 10.1074/jbc.M112.404434 PMC3493948

[52] Lytton J , Westlin M, Hanley MR. 1991. Thapsigargin inhibits the sarcoplasmic or endoplasmic reticulum Ca-ATPase family of calcium pumps. J Biol Chem. 266(26):17067–17071. 1832668

[53] Chen B , Mahaney JE, Mayer MU, Bigelow DJ, Squier TC. 2008. Concerted but noncooperative activation of nucleotide and actuator domains of the Ca-ATPase upon calcium binding. Biochemistry. 47(47):12448–12456. 18956892 10.1021/bi8014289 PMC3236681

[54] Espinoza-Fonseca LM , Thomas DD. 2011. Atomic-level characterization of the activation mechanism of SERCA by calcium. PLoS One. 6(10):e26936. 22046418 10.1371/journal.pone.0026936 PMC3203174

[55] Hou Z , et al 2012. 2-Color calcium pump reveals closure of the cytoplasmic headpiece with calcium binding. PLoS One. 7(7):e40369. 22808146 10.1371/journal.pone.0040369 PMC3394785

[56] Fearnley CJ , Roderick HL, Bootman MD. 2011. Calcium signaling in cardiac myocytes. Cold Spring Harb Perspect Biol. 3(11):a004242. 21875987 10.1101/cshperspect.a004242 PMC3220352

[57] Kirschenlohr HL , Grace AA, Vandenberg JI, Metcalfe JC, Smith GA. 2000. Estimation of systolic and diastolic free intracellular Ca2+ by titration of Ca2+ buffering in the ferret heart. Biochem J. 346 Pt 2(Pt 2):385–391. 10677357 PMC1220864

[58] Cheung JY , Tillotson DL, Yelamarty RV, Scaduto RC, Jr. 1989. Cytosolic free calcium concentration in individual cardiac myocytes in primary culture. Am J Physiol. 256(6 Pt 1):C1120–C1130. 2472066 10.1152/ajpcell.1989.256.6.C1120

[59] Thastrup O , Cullen PJ, Drobak BK, Hanley MR, Dawson AP. 1990. Thapsigargin, a tumor promoter, discharges intracellular Ca2+ stores by specific inhibition of the endoplasmic reticulum Ca2(+)-ATPase. Proc Natl Acad Sci U S A. 87(7):2466–2470. 2138778 10.1073/pnas.87.7.2466 PMC53710

[60] Kranias EG . 1985. Regulation of Ca2+ transport by cyclic 3',5'-AMP-dependent and calcium-calmodulin-dependent phosphorylation of cardiac sarcoplasmic reticulum. Biochim Biophys Acta. 844(2):193–199. 2982423 10.1016/0167-4889(85)90090-4

[61] Ohmori F , Tada M, Kinoshita N, Matsuo H, Sakakibara H. 1976. Effect of protein kinase modulator on cAMP-dependent protein kinase-catalyzed phosphorylation of phospholamban and stimulation of calcium transport in cardiac sarcoplasmic reticulum. Recent Adv Stud Cardiac Struct Metab. 11:279–284. 201986

[62] Akin BL , Jones LR. 2012. Characterizing phospholamban to sarco(endo)plasmic reticulum ca2+-ATPase 2a (SERCA2a) protein binding interactions in human cardiac sarcoplasmic reticulum vesicles using chemical cross-linking. J Biol Chem. 287(10):7582–7593. 22247554 10.1074/jbc.M111.334987 PMC3293560

[63] Chen Z , Akin BL, Stokes DL, Jones LR. 2006. Cross-linking of C-terminal residues of phospholamban to the Ca2+ pump of cardiac sarcoplasmic reticulum to probe spatial and functional interactions within the transmembrane domain. J Biol Chem. 281(20):14163–14172. 16554295 10.1074/jbc.M601338200

[64] Toyoshima C , et al 2003. Modeling of the inhibitory interaction of phospholamban with the Ca2+ ATPase. Proc Natl Acad Sci USA. 100(2):467–472. 12525698 10.1073/pnas.0237326100 PMC141018

[65] Zamoon J , Nitu F, Karim C, Thomas DD, Veglia G. 2005. Mapping the interaction surface of a membrane protein: unveiling the conformational switch of phospholamban in calcium pump regulation. Proc Natl Acad Sci USA. 102(13):4747–4752. 15781867 10.1073/pnas.0406039102 PMC555693

[66] Asahi M , Kimura Y, Kurzydlowski K, Tada M, MacLennan DH. 1999. Transmembrane helix M6 in sarco(endo)plasmic reticulum Ca(2+)-ATPase forms a functional interaction site with phospholamban. Evidence for physical interactions at other sites. J Biol Chem. 274(46):32855–32862. 10551848 10.1074/jbc.274.46.32855

[67] Morita T , et al 2008. Interaction sites among phospholamban, sarcolipin, and the sarco(endo)plasmic reticulum Ca(2+)-ATPase. Biochem Biophys Res Commun. 369(1):188–194. 18053795 10.1016/j.bbrc.2007.11.098

[68] Akin BL , Hurley TD, Chen Z, Jones LR. 2013. The structural basis for phospholamban inhibition of the calcium pump in sarcoplasmic reticulum. J Biol Chem. 288(42):30181–30191. 23996003 10.1074/jbc.M113.501585 PMC3798486

[69] Espinoza-Fonseca LM , Autry JM, Ramirez-Salinas GL, Thomas DD. 2015. Atomic-level mechanisms for phospholamban regulation of the calcium pump. Biophys J. 108(7):1697–1708. 25863061 10.1016/j.bpj.2015.03.004 PMC4390807

[70] Espinoza-Fonseca LM , Autry JM, Thomas DD. 2015. Sarcolipin and phospholamban inhibit the calcium pump by populating a similar metal ion-free intermediate state. Biochem Biophys Res Commun. 463(1–2):37–41. 25983321 10.1016/j.bbrc.2015.05.012 PMC4465059

[71] Asahi M , McKenna E, Kurzydlowski K, Tada M, MacLennan DH. 2000. Physical interactions between phospholamban and sarco(endo)plasmic reticulum Ca^2+^-ATPases are dissociated by elevated Ca^2+^, but not by phospholamban phosphorylation, vanadate, or thapsigargin, and are enhanced by ATP. J Biol Chem. 275(20):15034–15038. 10809745 10.1074/jbc.275.20.15034

[72] Chen Z , Akin BL, Jones LR. 2010. Ca^2+^ binding to site I of the cardiac Ca^2+^ pump is sufficient to dissociate phospholamban. J Biol Chem. 285(5):3253–3260. 19948724 10.1074/jbc.M109.080820 PMC2823463

[73] Chen Z . 2015. Role of nucleotides in stabilization of the phospholamban/cardiac Ca(2)(+) pump inhibitory complex examined with use of metal fluorides. FEBS J. 282(22):4402–4414. 26337774 10.1111/febs.13506

[74] Pallikkuth S , et al 2013. Phosphorylated phospholamban stabilizes a compact conformation of the cardiac calcium-ATPase. Biophys J. 105(8):1812–1821. 24138857 10.1016/j.bpj.2013.08.045 PMC3797577

[75] Krasteva M , Barth A. 2007. Structures of the Ca2+-ATPase complexes with ATP, AMPPCP and AMPPNP. An FTIR study. Biochim Biophys Acta. 1767(1):114–123. 17157262 10.1016/j.bbabio.2006.11.003

[76] Kast D , Espinoza-Fonseca LM, Yi C, Thomas DD. 2010. Phosphorylation-induced structural changes in smooth muscle myosin regulatory light chain. Proc Natl Acad Sci USA. 107(18):8207–8212. 20404208 10.1073/pnas.1001941107 PMC2889560

